# Supplementary material for: Diversity in root growth responses to moisture deficit in young faba bean (Vicia faba L.) plants
Source: PeerJ. 2018 Feb 21;6:e4401. doi: 10.7717/peerj.4401 (PMC5826991; doi:10.7717/peerj.4401)
Supplement: Table S1 — *, **, *** p < 0.05, 0.01, 0.001, respectively. [file peerj-06-4401-s001.docx]

| **Accessions** | **Stomatal Conductance mmol H2O/m2/s** | **Rank (smallest to largest)** | **Leaf surface temperature (°C)** | **Rank (smallest to largest)** | **SPAD value** | **Rank (largest to smallest)** | **Shoot dry weight (g)** | **Rank(largest to smallest)** | **Root dry weight (g)** | **Rank (largest to smallest)** | **Root: shoot dry weight ratio** | **Rank (largest to smallest)** | **Root mass fraction** | **Rank (largest to smallest)** | **Sum of ranks** |
| --- | --- | --- | --- | --- | --- | --- | --- | --- | --- | --- | --- | --- | --- | --- | --- |
| WS124242 | 109 | 1 | 21.3 | 7 | 38 | 4 | 1.61 | 17 | 0.96 | 7 | 0.60 | 3 | 0.39 | 6 | 45 |
| DS13463 | 155 | 4 | 20.9 | 3 | 33 | 9 | 2.26 | 10 | 0.89 | 8 | 0.40 | 5 | 0.29 | 16 | 55 |
| DS13481 | 201 | 7 | 21.1 | 5 | 34 | 8 | 2.52 | 8 | 1.03 | 7 | 0.41 | 5 | 0.30 | 15 | 55 |
| WS117868 | 230 | 13 | 20.8 | 2 | 33 | 9 | 2.45 | 8 | 0.88 | 8 | 0.36 | 5 | 0.27 | 18 | 63 |
| DS72309 | 237 | 15 | 20.9 | 3 | 33 | 9 | 2.41 | 9 | 0.98 | 7 | 0.41 | 5 | 0.30 | 15 | 63 |
| DS11210 | 242 | 16 | 22.4 | 17 | 39 | 3 | 2.64 | 7 | 1.31 | 4 | 0.50 | 4 | 0.33 | 12 | 63 |
| DS11437 | 251 | 19 | 20.7 | 1 | 34 | 8 | 1.88 | 14 | 0.97 | 7 | 0.51 | 4 | 0.34 | 11 | 64 |
| DS131708 | 153 | 3 | 22.2 | 15 | 35 | 7 | 0.53 | 27 | 0.37 | 13 | 0.70 | 2 | 0.45 | 2 | 69 |
| WS99379 | 236 | 14 | 22.1 | 14 | 36 | 6 | 2.43 | 9 | 1.03 | 7 | 0.42 | 5 | 0.30 | 15 | 70 |
| DS72387 | 203 | 9 | 23.0 | 22 | 39 | 3 | 1.31 | 19 | 0.69 | 10 | 0.53 | 4 | 0.41 | 4 | 71 |
| DS112096 | 215 | 11 | 21.9 | 12 | 33 | 9 | 1.79 | 15 | 0.86 | 8 | 0.48 | 4 | 0.33 | 12 | 71 |
| Messay | 221 | 12 | 21.6 | 10 | 34 | 8 | 1.99 | 13 | 0.80 | 9 | 0.40 | 5 | 0.29 | 16 | 73 |
| DS11207 | 281 | 25 | 21.3 | 7 | 33 | 9 | 2.28 | 10 | 1.08 | 6 | 0.47 | 4 | 0.32 | 13 | 74 |
| DS72523 | 180 | 6 | 22.6 | 18 | 36 | 6 | 1.50 | 18 | 0.62 | 11 | 0.41 | 5 | 0.33 | 12 | 76 |
| WS99465 | 202 | 8 | 22.2 | 15 | 30 | 12 | 2.09 | 12 | 0.82 | 9 | 0.39 | 5 | 0.29 | 16 | 77 |
| DS11561 | 282 | 26 | 22.0 | 13 | 38 | 4 | 2.55 | 8 | 1.07 | 6 | 0.42 | 5 | 0.30 | 15 | 77 |
| DS11286 | 299 | 34 | 22.1 | 14 | 34 | 8 | 2.91 | 5 | 1.47 | 2 | 0.51 | 4 | 0.34 | 11 | 78 |
| EH 06006-6 | 155 | 4 | 24.1 | 31 | 37 | 5 | 2.97 | 4 | 0.93 | 8 | 0.31 | 6 | 0.24 | 21 | 79 |
| WS11309 | 273 | 24 | 21.9 | 12 | 34 | 8 | 2.07 | 12 | 1.05 | 7 | 0.51 | 4 | 0.33 | 12 | 79 |
| DS72493 | 314 | 37 | 21.0 | 4 | 41 | 1 | 2.01 | 13 | 0.97 | 7 | 0.48 | 4 | 0.32 | 13 | 79 |
| WS117864 | 161 | 5 | 23.4 | 26 | 28 | 14 | 2.27 | 10 | 1.00 | 7 | 0.44 | 5 | 0.31 | 14 | 81 |
| DS70622 | 330 | 42 | 21.7 | 11 | 33 | 9 | 3.49 | 1 | 1.59 | 1 | 0.46 | 4 | 0.31 | 14 | 82 |
| Tesfa | 248 | 18 | 21.7 | 11 | 34 | 8 | 1.71 | 16 | 0.67 | 10 | 0.39 | 5 | 0.29 | 16 | 84 |
| DS12257 | 291 | 31 | 22.4 | 17 | 39 | 3 | 2.17 | 11 | 1.06 | 6 | 0.49 | 4 | 0.33 | 12 | 84 |
| Babylon | 305 | 36 | 20.8 | 2 | 35 | 7 | 1.12 | 21 | 0.67 | 10 | 0.60 | 3 | 0.38 | 7 | 86 |
| DS11202 | 145 | 2 | 24.4 | 32 | 29 | 13 | 2.63 | 7 | 0.90 | 8 | 0.34 | 6 | 0.26 | 19 | 87 |
| Kassa | 205 | 10 | 22.6 | 18 | 30 | 12 | 1.01 | 22 | 0.50 | 12 | 0.49 | 4 | 0.34 | 11 | 89 |
| DS74370 | 296 | 32 | 22.0 | 13 | 38 | 4 | 2.31 | 10 | 0.91 | 8 | 0.39 | 5 | 0.28 | 17 | 89 |
| WS13107 | 288 | 30 | 23.2 | 24 | 37 | 5 | 2.99 | 4 | 1.14 | 6 | 0.38 | 5 | 0.29 | 16 | 90 |
| Aurora | 335 | 45 | 21.3 | 7 | 35 | 7 | 1.34 | 19 | 0.95 | 8 | 0.71 | 2 | 0.41 | 4 | 92 |
| DS11294 | 324 | 39 | 21.4 | 8 | 33 | 9 | 1.56 | 17 | 0.86 | 8 | 0.55 | 4 | 0.37 | 8 | 93 |
| WS12315 | 244 | 17 | 22.2 | 15 | 28 | 14 | 0.88 | 23 | 0.50 | 12 | 0.56 | 3 | 0.35 | 10 | 94 |
| WS115177 | 271 | 23 | 21.7 | 11 | 29 | 13 | 0.69 | 25 | 0.43 | 13 | 0.62 | 3 | 0.39 | 6 | 94 |
| WS132266 | 287 | 29 | 22.1 | 14 | 33 | 9 | 2.10 | 12 | 0.81 | 9 | 0.39 | 5 | 0.28 | 17 | 95 |
| DS72366 | 333 | 44 | 22.1 | 14 | 40 | 2 | 2.70 | 6 | 1.05 | 7 | 0.39 | 5 | 0.28 | 17 | 95 |
| WS130600 | 303 | 35 | 21.3 | 7 | 35 | 7 | 1.93 | 14 | 0.69 | 10 | 0.36 | 5 | 0.27 | 18 | 96 |
| DS72455 | 303 | 35 | 22.8 | 20 | 36 | 6 | 3.12 | 3 | 1.05 | 6 | 0.34 | 6 | 0.25 | 20 | 96 |
| WS130731 | 315 | 38 | 20.9 | 3 | 33 | 9 | 1.93 | 14 | 0.74 | 10 | 0.38 | 5 | 0.28 | 17 | 96 |
| DS74573 | 332 | 43 | 22.6 | 18 | 30 | 12 | 3.38 | 2 | 1.41 | 3 | 0.42 | 5 | 0.30 | 15 | 98 |
| WS132274 | 298 | 33 | 21.9 | 12 | 28 | 14 | 2.64 | 7 | 0.91 | 8 | 0.35 | 6 | 0.26 | 19 | 99 |
| WS132238 | 284 | 27 | 22.3 | 16 | 29 | 13 | 1.94 | 14 | 0.76 | 9 | 0.39 | 5 | 0.29 | 16 | 100 |
| WS11344 | 342 | 47 | 22.0 | 13 | 36 | 6 | 1.74 | 16 | 0.99 | 7 | 0.57 | 3 | 0.36 | 9 | 101 |
| Melodie/2 | 252 | 20 | 23.3 | 25 | 39 | 3 | 0.74 | 25 | 0.38 | 13 | 0.51 | 4 | 0.32 | 13 | 103 |
| DS72271 | 264 | 22 | 23.6 | 28 | 32 | 10 | 1.81 | 15 | 0.80 | 9 | 0.44 | 5 | 0.31 | 14 | 103 |
| WS11313 | 303 | 35 | 22.1 | 14 | 32 | 10 | 1.85 | 14 | 0.76 | 9 | 0.41 | 5 | 0.29 | 16 | 103 |
| DS11909 | 343 | 48 | 21.3 | 7 | 30 | 12 | 2.43 | 9 | 0.96 | 7 | 0.39 | 5 | 0.29 | 16 | 104 |
| WS117855 | 314 | 37 | 22.0 | 13 | 28 | 14 | 2.43 | 9 | 0.87 | 8 | 0.36 | 5 | 0.26 | 19 | 105 |
| WS114476 | 256 | 21 | 23.9 | 29 | 30 | 12 | 0.27 | 29 | 0.25 | 14 | 0.94 | 1 | 0.50 | 1 | 107 |
| WS115134 | 287 | 29 | 22.2 | 15 | 35 | 7 | 0.37 | 28 | 0.22 | 15 | 0.59 | 3 | 0.35 | 10 | 107 |
| GLA 1103 | 314 | 37 | 22.6 | 18 | 36 | 6 | 1.20 | 20 | 0.62 | 11 | 0.52 | 4 | 0.34 | 11 | 107 |
| DS13473 | 380 | 57 | 22.0 | 13 | 34 | 8 | 2.33 | 10 | 1.21 | 5 | 0.52 | 4 | 0.35 | 10 | 107 |
| DS99515 | 394 | 60 | 22.1 | 14 | 37 | 5 | 1.91 | 14 | 1.19 | 5 | 0.62 | 3 | 0.39 | 6 | 107 |
| DS72310 | 285 | 28 | 22.7 | 19 | 32 | 10 | 1.69 | 16 | 0.57 | 11 | 0.34 | 6 | 0.26 | 19 | 109 |
| NC 58 | 287 | 29 | 24.0 | 30 | 33 | 9 | 2.14 | 12 | 0.90 | 8 | 0.42 | 5 | 0.29 | 16 | 109 |
| DS124353 | 326 | 40 | 22.2 | 15 | 33 | 9 | 1.76 | 15 | 0.74 | 10 | 0.42 | 5 | 0.30 | 15 | 109 |
| WS117849 | 327 | 41 | 22.2 | 15 | 24 | 17 | 3.06 | 3 | 0.98 | 7 | 0.32 | 6 | 0.25 | 20 | 109 |
| WS117830 | 305 | 36 | 22.0 | 13 | 29 | 13 | 2.08 | 12 | 0.69 | 10 | 0.33 | 6 | 0.25 | 20 | 110 |
| DS11317 | 336 | 46 | 23.0 | 22 | 33 | 9 | 2.46 | 8 | 1.07 | 6 | 0.43 | 5 | 0.31 | 14 | 110 |
| WS117857 | 303 | 35 | 22.9 | 21 | 27 | 15 | 2.07 | 12 | 0.80 | 9 | 0.39 | 5 | 0.29 | 16 | 113 |
| WS13185 | 444 | 68 | 22.1 | 14 | 36 | 6 | 1.78 | 15 | 1.20 | 5 | 0.68 | 2 | 0.41 | 4 | 114 |
| DS11236 | 412 | 62 | 22.6 | 18 | 36 | 6 | 2.56 | 7 | 1.19 | 5 | 0.46 | 4 | 0.32 | 13 | 115 |
| DS124062 | 343 | 48 | 22.7 | 19 | 29 | 13 | 1.67 | 16 | 0.72 | 10 | 0.43 | 5 | 0.39 | 6 | 117 |
| DS124138 | 381 | 58 | 21.1 | 5 | 35 | 7 | 1.23 | 20 | 0.62 | 11 | 0.50 | 4 | 0.33 | 12 | 117 |
| DS11591 | 360 | 53 | 22.3 | 16 | 31 | 11 | 1.51 | 18 | 0.84 | 9 | 0.55 | 3 | 0.37 | 8 | 118 |
| WS132258 | 354 | 50 | 23.1 | 23 | 29 | 13 | 2.19 | 11 | 1.08 | 6 | 0.49 | 4 | 0.33 | 12 | 119 |
| DS13042 | 354 | 50 | 23.4 | 26 | 34 | 8 | 1.45 | 18 | 0.70 | 10 | 0.48 | 4 | 0.43 | 3 | 119 |
| ILB938/2 | 383 | 59 | 21.7 | 11 | 36 | 6 | 1.92 | 14 | 0.84 | 9 | 0.44 | 5 | 0.30 | 15 | 119 |
| DS74554 | 346 | 49 | 22.4 | 17 | 35 | 7 | 2.16 | 11 | 0.75 | 10 | 0.35 | 6 | 0.25 | 20 | 120 |
| DS11788 | 398 | 61 | 21.5 | 9 | 34 | 8 | 1.64 | 17 | 0.77 | 9 | 0.47 | 4 | 0.33 | 12 | 120 |
| DS11320 | 752 | 73 | 21.2 | 6 | 26 | 16 | 2.65 | 7 | 1.30 | 4 | 0.49 | 4 | 0.34 | 11 | 121 |
| DS11281 | 421 | 66 | 22.1 | 14 | 33 | 9 | 2.08 | 12 | 1.05 | 6 | 0.50 | 4 | 0.34 | 11 | 122 |
| DS11689 | 360 | 53 | 22.7 | 19 | 39 | 3 | 1.23 | 20 | 0.57 | 11 | 0.46 | 4 | 0.32 | 13 | 123 |
| DOSHA | 369 | 54 | 21.9 | 12 | 32 | 10 | 1.62 | 17 | 0.65 | 10 | 0.40 | 5 | 0.29 | 16 | 124 |
| DS13918 | 370 | 55 | 22.5 | 18 | 37 | 5 | 1.77 | 15 | 0.72 | 10 | 0.40 | 5 | 0.29 | 16 | 124 |
| DS11701 | 372 | 56 | 22.5 | 18 | 35 | 7 | 0.72 | 25 | 0.46 | 12 | 0.64 | 3 | 0.40 | 5 | 126 |
| WS11688 | 394 | 60 | 21.2 | 6 | 30 | 12 | 0.96 | 22 | 0.45 | 13 | 0.46 | 4 | 0.34 | 11 | 128 |
| DS11480 | 414 | 63 | 22.0 | 13 | 35 | 7 | 1.91 | 14 | 0.73 | 10 | 0.38 | 5 | 0.28 | 17 | 129 |
| WS13039 | 467 | 72 | 21.0 | 4 | 29 | 13 | 1.02 | 22 | 0.54 | 12 | 0.52 | 4 | 0.40 | 5 | 132 |
| WS99501 | 357 | 51 | 23.0 | 22 | 32 | 10 | 1.66 | 16 | 0.75 | 10 | 0.45 | 5 | 0.26 | 19 | 133 |
| WS117853 | 415 | 64 | 22.5 | 18 | 31 | 11 | 2.06 | 12 | 0.88 | 8 | 0.43 | 5 | 0.30 | 15 | 133 |
| DS137675 | 420 | 65 | 21.5 | 9 | 33 | 9 | 1.22 | 20 | 0.49 | 12 | 0.40 | 5 | 0.29 | 16 | 136 |
| WS115182 | 359 | 52 | 22.1 | 14 | 28 | 14 | 0.36 | 28 | 0.21 | 15 | 0.58 | 3 | 0.34 | 11 | 137 |
| WS114576 | 354 | 50 | 22.0 | 13 | 28 | 14 | 0.45 | 28 | 0.23 | 15 | 0.51 | 4 | 0.31 | 14 | 138 |
| Gebelcho | 360 | 53 | 24.8 | 33 | 34 | 8 | 2.23 | 11 | 0.80 | 9 | 0.36 | 5 | 0.26 | 19 | 138 |
| DS72396 | 424 | 67 | 22.7 | 19 | 35 | 7 | 0.81 | 24 | 0.49 | 12 | 0.60 | 3 | 0.38 | 7 | 139 |
| WS115430 | 446 | 69 | 21.1 | 5 | 29 | 13 | 0.58 | 26 | 0.24 | 15 | 0.42 | 5 | 0.30 | 15 | 148 |
| WS115352 | 394 | 60 | 22.9 | 21 | 29 | 13 | 0.43 | 28 | 0.21 | 15 | 0.49 | 4 | 0.36 | 9 | 150 |
| WS115186 | 449 | 70 | 21.6 | 10 | 27 | 15 | 0.41 | 28 | 0.22 | 15 | 0.53 | 4 | 0.32 | 13 | 155 |
| WS117841 | 457 | 71 | 23.5 | 27 | 28 | 14 | 1.76 | 15 | 0.71 | 10 | 0.40 | 5 | 0.29 | 16 | 158 |
| **SE** | 65 |  | 0.7 |  | 2 |  | 0.27 |  | 0.12 |  | 0.13 |  | 0.04 |  |  |
| **LSD (5%)** | 182 |  | 2.0 |  | 4 |  | 0.75 |  | 0.35 |  | 0.37 |  | 0.10 |  |  |
| P-value | *** |  | * |  | *** |  | *** |  | *** |  | ** |  | *** |  |  |
