# Supplementary material for: Diversity in root growth responses to moisture deficit in young faba bean (Vicia faba L.) plants
Source: PeerJ. 2018 Feb 21;6:e4401. doi: 10.7717/peerj.4401 (PMC5826991; doi:10.7717/peerj.4401)
Supplement: Table S2 — *, **, *** p < 0.05, 0.01, 0.001, respectively. [file peerj-06-4401-s002.docx]

| Accessions | Tap root length (cm) | | | Lateral root length (cm) | | Second order lateral root length (cm) | |
| --- | --- | --- | --- | --- | --- | --- | --- |
|  | 5 DAT | 12 DAT | | 5 DAT | 12 DAT | 12 DAT | |
|  | Mean value | Well watered | Water limited | Mean value | Mean value | Well watered | Water limited |
| DS11202 | 15.9ab | 46.3bc | 17.7ab | 38a | 148abc | 6ab | 4.9 |
| DS11320 | 25.2bc | 63.5d | 32.2bc | 102bc | 305cd | 18abc | 0.5 |
| DS70622 | 19.8abc | 50.7cd | 34.9c | 137c | 403d | 33c | 0.0 |
| DS74573 | 18.5abc | 52.6cd | 16.5a | 71ab | 272cd | 0a | 2.9 |
| EH06006-6 | 26.8c | 59.4cd | 27.2abc | 116bc | 262bcd | 1ab | 0.0 |
| ILB938/2 | 20.2abc | 45.5bc | 17.9ab | 109bc | 248abcd | 4ab | 1.7 |
| Melodie/2 | 10.6a | 28.3a | 22.8abc | 25a | 96a | 2ab | 0.6 |
| WS99501 | 11.2a | 35.6ab | 16.8a | 34a | 107ab | 21bc | 0.3 |
| SE | 2.2 | 4.6 | | 16 | 35 | 3.2 | |
| LSD (5%) | 6.3 | 13.1 | | 46 | 100 | 9.2 | |
| **Treatment** |  |  |  |  |  |  |  |
| Well watered | 23.7 | 47.8 | | 106 | 326 | 10.5 | |
| Water limited | 13.3 | 23.2 | | 51 | 134 | 1.4 | |
| SE | 1.1 | 1.6 | | 8 | 18 | 1.6 | |
| LSD (5%) | 3.2 | 4.6 | | 23 | 50 | 4.6 | |
| **P-value** |  |  |  |  |  |  |  |
| Treatment | ** | ** | | * | * | * | |
| Accession | *** | *** | | *** | *** | ** | |
| Treatment x Accession | ns | * | | ns | ns | ** | |
