# Supplementary material for: Diversity in root growth responses to moisture deficit in young faba bean (Vicia faba L.) plants
Source: PeerJ. 2018 Feb 21;6:e4401. doi: 10.7717/peerj.4401 (PMC5826991; doi:10.7717/peerj.4401)
Supplement: Table S3 — *, **, *** p < 0.05, 0.01, 0.001, respectively. SE is standard error and LSD is least significant difference. [file peerj-06-4401-s003.docx]

| Accessions | Total root length (cm) | | Root system depth (cm) | | Root system width (cm) | | Convex hull area (cm^2^) | |
| --- | --- | --- | --- | --- | --- | --- | --- | --- |
|  | 5 DAT | 12 DAT | 5 DAT | 12 DAT | 5 DAT | 12 DAT | 5 DAT | 12 DAT |
| DS11202 | 53a | 185ab | 17abc | 35abc | 9a | 22ab | 91ab | 503ab |
| DS11320 | 127b | 361bc | 28d | 48c | 14abc | 31bc | 224bcd | 894bc |
| DS70622 | 158b | 462c | 23bcd | 46bc | 14abc | 27abc | 215bcd | 825abc |
| DS74573 | 89ab | 308abc | 22bcd | 40abc | 12ab | 29abc | 163abc | 729abc |
| EH06006-6 | 143b | 306abc | 30d | 47c | 19c | 35c | 332d | 1041c |
| ILB938/2 | 131b | 283abc | 25cd | 36abc | 18bc | 29abc | 256cd | 670abc |
| Melodie/2 | 35a | 123a | 12a | 30ab | 8a | 21ab | 52a | 403ab |
| WS99501 | 45a | 144a | 13ab | 29a | 9a | 18a | 83ab | 361a |
| SE | 18 | 37 | 2 | 3 | 2 | 3 | 31 | 96 |
| LSD (5%) | 51 | 106 | 6 | 8 | 5 | 9 | 87 | 275 |
| **Treatment** |  |  |  |  |  |  |  |  |
| Well watered | 130 | 384 | 26 | 50 | 16 | 32 | 251 | 1001 |
| Water limited | 65 | 159 | 16 | 27 | 10 | 21 | 103 | 355 |
| SE | 9 | 19 | 1 | 1 | 1 | 1 | 15 | 48 |
| LSD (5%) | 26 | 53 | 3 | 4 | 2 | 4 | 44 | 137 |
| **P-value** |  |  |  |  |  |  |  |  |
| Treatment | * | * | ** | ** | ns | * | * | ** |
| Accession | *** | *** | *** | *** | *** | ** | *** | *** |
| Treatment x Accession | ns | ns | ns | ns | ns | ns | ns | ns |
