# Supplementary material for: Diversity in root growth responses to moisture deficit in young faba bean (Vicia faba L.) plants
Source: PeerJ. 2018 Feb 21;6:e4401. doi: 10.7717/peerj.4401 (PMC5826991; doi:10.7717/peerj.4401)
Supplement: Table S6 — **, *** p < 0.01, 0.001, respectively. df is degrees of freedom. [file peerj-06-4401-s006.docx]

| **Source of variation** | **df** | **Mean square** | | | | | | | | | | | | | |
| --- | --- | --- | --- | --- | --- | --- | --- | --- | --- | --- | --- | --- | --- | --- | --- |
|  |  | **Stomatal conductance** | | **Leaf surface temperature** | | **Chlorophyll concentration (SPAD value)** | | **Shoot dry weight** | | **Root dry weight** | | **Root to shoot dry weight ratio** | | **Root mass fraction** | |
| Block | 3 | 3483678 | *** | 20.41 | *** | 272.76 | *** | 14.060 | *** | 1.276 | *** | 0.8653 | *** | 0.1230 | *** |
| Accession | 88 | 33633 | *** | 2.918 | * | 52.22 | *** | 2.195 | ** | 0.3477 | *** | 0.1023 | ** | 0.00990 | *** |
| Error | 264 | 17045 |  | 2.141 |  | 9.774 |  | 0.293 |  | 0.062 |  | 0.069 |  | 0.005 |  |
| **R^2^ due to Block, %** | | 58.5 |  | 6.9 |  | 10.2 |  | 13.5 |  | 7.5 |  | 8.7 |  | 14.5 |  |
| Block 1 |  | 111.1 |  | 22.6 |  | 35.7 |  | 1.3 |  | 0.65 |  | 0.58 |  | 0.34 |  |
| Block 2 |  | 410.9 |  | 21.5 |  | 32.4 |  | 2.0 |  | 0.77 |  | 0.39 |  | 0.28 |  |
| Block 3 |  | 544.2 |  | 22.2 |  | 32.2 |  | 2.2 |  | 0.90 |  | 0.46 |  | 0.31 |  |
| Block 4 |  | 196.1 |  | 22.4 |  | 32.0 |  | 1.7 |  | 0.90 |  | 0.60 |  | 0.36 |  |
